# Global Health Immersions in Nursing Education: Evaluating Impact and Recommendations for Programming

**DOI:** 10.1111/jan.70188

**Published:** 2025-08-29

**Authors:** Claire Honl, Katherine Collins

**Affiliations:** ^1^ School of Nursing University of Wisconsin‐Madison Madison Wisconsin USA

**Keywords:** global health, graduate, nursing education, undergraduate

## Abstract

**Aim:**

Global health immersion programming is cited as supporting nursing students' cultural competency; it is also historically grounded in colonialism. This study explored nursing students' perspectives on the benefits and challenges of global health immersions. Programming suggestions are based on student feedback and immersion literature.

**Design:**

Qualitative, cross‐sectional.

**Methods:**

Nine semi‐structured interviews were conducted with nursing students who participated in global health immersions. Interviews were inductively coded for recurring themes, focusing on immersion benefits, challenges, and impact on nursing practice.

**Results:**

Participants reported enriched cultural humility, deepened integration into local healthcare systems, and enhanced understandings of disparities. In clinical practice, participants described being galvanised to provide culturally respectful care, strengthening communication, valuing holistic care, desiring to promote health equity, and appreciating resources. Challenges included communication barriers, ethical concerns, and insufficient preparatory guidance. Challenges and suggestions for improvement are aligned with evidence‐based recommendations for ethical immersion programming.

**Conclusion:**

This study highlights the potential of immersions to advance culturally sensitive, equity‐centred healthcare. It identifies areas for growth and solutions to challenges through evidence‐based recommendations. Future research should invite feedback from collaborating international communities to understand programme impact and ensure mutual benefit.

**Reporting Method:**

SRQR guidelines.

**Patient/Public Contribution:**

No patient or public involvement in study design, conduct, or reporting.

**Summary**

- Implications for the profession/patient care
  ○ This work informs the optimisation of immersions to shape culturally respectful and equity‐driven nurses.
- Impact
  ○ It is crucial to assess the utility of global health immersions for participants and their clinical practice. While immersion programs offer transformative experiences, they require thoughtful planning and stakeholder collaboration, especially with international communities. Nursing educators should leverage findings to advance healthcare equity and support immersion decolonisation.
- Clinical community contributions
  ○ Highlights student perspectives on immersions and domains for improvement in global health collaborations.
  ○ Provides recommendations for optimising nursing global health curriculum.

## 1 Introduction

Following the COVID‐19 pandemic, there is a forecasted heightened demand for global health immersion (GHI) programming and an increased emphasis on global health education (Jacobsen and Waggett 2022). Further, GHI programs are cited as tools to promote cultural humility and respect, concepts that are increasing priorities within the nursing and healthcare domains (Kasper et al. 2020; Hughes et al. 2020; Foronda 2020). With GHI programs proliferating rapidly, especially in high‐income countries, there is a need to critically analyse the programs' impacts on all stakeholders (Kasper et al. 2020).

While not a requirement of GHIs, many such programs involve collaboration with an international community that may be less well‐resourced than the community of the visiting institution. In these cases, the GHI may be presented as a “mission” or “charity” trip, as well as an educational opportunity for student participants. There is a risk of unintended negative consequences posed to collaborating international communities, and trips can become more focused on “volun‐tourism” rather than the mutual benefit of all stakeholders (Zientek and Bonnell 2020; Sullivan 2019; McKinley Yoder et al. 2022; Advocacy for Global Health Partnerships n.d.).

The literature suggests that GHIs carry both risks and benefits, and evaluation of GHI participant experiences is encouraged in the guidelines set forth by the Working Group on Ethics Guidelines for Global Health Training (WEIGHT) (Crump et al. 2010). Therefore, it is important for institutions promoting GHI programmes to elicit feedback from GHI participants and identify areas of improvement within the programmes it offers.

## 2 Background

Following the post‐pandemic return of institutional GHI programs, the study team sought to clarify the impact of program participation on future and current nurses. The study institution had no recent prior GHI program evaluation that offered the depth and breadth of insight inherent to qualitative interviewing. Recognising the ethical dilemmas inherent to global health immersion trips, the team acknowledged a need to evaluate these trips' utility and impact both on trip participants and the international communities that collaborate with students and GHI faculty (McKinley Yoder et al. 2022). It is important to acknowledge that resource limitations in place at the time of the study prohibited eliciting feedback from international collaborators; however, collaborators' voices are crucial to assess GHI impact and necessary for a comprehensive program evaluation. The feedback elicited in this initiative represents a sub‐set of the institution's GHI stakeholders.

Previous literature exists on impacts of GHI programming; however, there is a paucity of research that incorporates both the graduate and undergraduate perspectives. Additionally, this initiative was unique in that student participants were interviewed by a fellow student peer, lessening the power differential and encouraging participants to speak freely. This report summarises student feedback on GHI strengths, challenges, and impacts on clinical practice. The study aligns students' challenges and recommendations with evidence‐based best practices for GHI improvement.

## 3 The Study

The study aimed to understand the benefits and challenges associated with GHI programs and identify opportunities to strengthen GHI for undergraduate and graduate nursing students at the institution. Student feedback was integrated with literature defining ethical immersion practices, and specific focus was paid to the impact of GHI participation on current and/or future nursing practice. Overall, the initiative strove to answer the question: What benefits and challenges do GHI participants encounter, and what is the impact of GHI participation on current and future nurses?

## 4 Methods/Methodology

A qualitative descriptive design was selected for the initiative to make meaning of participants' GHI experiences and leverage the experiences to improve GHI programming in the context of quality improvement (QI) (Doyle et al. 2019).

The study team contacted graduate and undergraduate nursing students who participated in GHI programming during 2023 requesting a 30 to 60 min interview. Nine one‐on‐one interviews were conducted virtually by a graduate nursing student with previous qualitative interviewing experience and training. Six interviews were conducted with graduate students and three interviews were conducted with undergraduate students. The interviewer used a semi‐structured interview guide centred on the topics of GHI strengths, weaknesses, and impacts on clinical practice. Students were encouraged to speak freely and reminded that their responses would remain confidential and de‐identified outside of the study team.

Interviews were transcribed verbatim using video‐transcription technology and manually reviewed and revised for accuracy. Transcripts were also de‐identified with identifying details omitted. Using grounded theory, a subset of interviews was inductively assessed for recurring themes, and a codebook was developed in Excel. All interviews were then coded by hand, and the codebook was iteratively revised as new topics emerged. Finally, code queries were created and coded content further analysed for respondent themes. Coding was reviewed by all members of the team to ensure consistent and reliable code application. Analysis was completed when thematic saturation was reached.

### 4.1 Ethical Considerations

This study was conducted as part of a quality improvement initiative evaluating global health immersion programming. Institutional Review Board (IRB) oversight was not required, as the project focused on program evaluation for the purposes of institutional‐level improvements. The institutional IRB evaluation tool was utilised, and the project was deemed a quality improvement program evaluation. Participants provided explicit consent prior to participation; participants were also informed of their right to refuse questions or opt out of the interview at any time. Finally, explicit consent to disseminate deidentified participant feedback was also obtained, and all data was de‐identified to ensure participant anonymity outside of the study team.

## 5 Findings

### 5.1 Strengths

**FIGURE 1:**
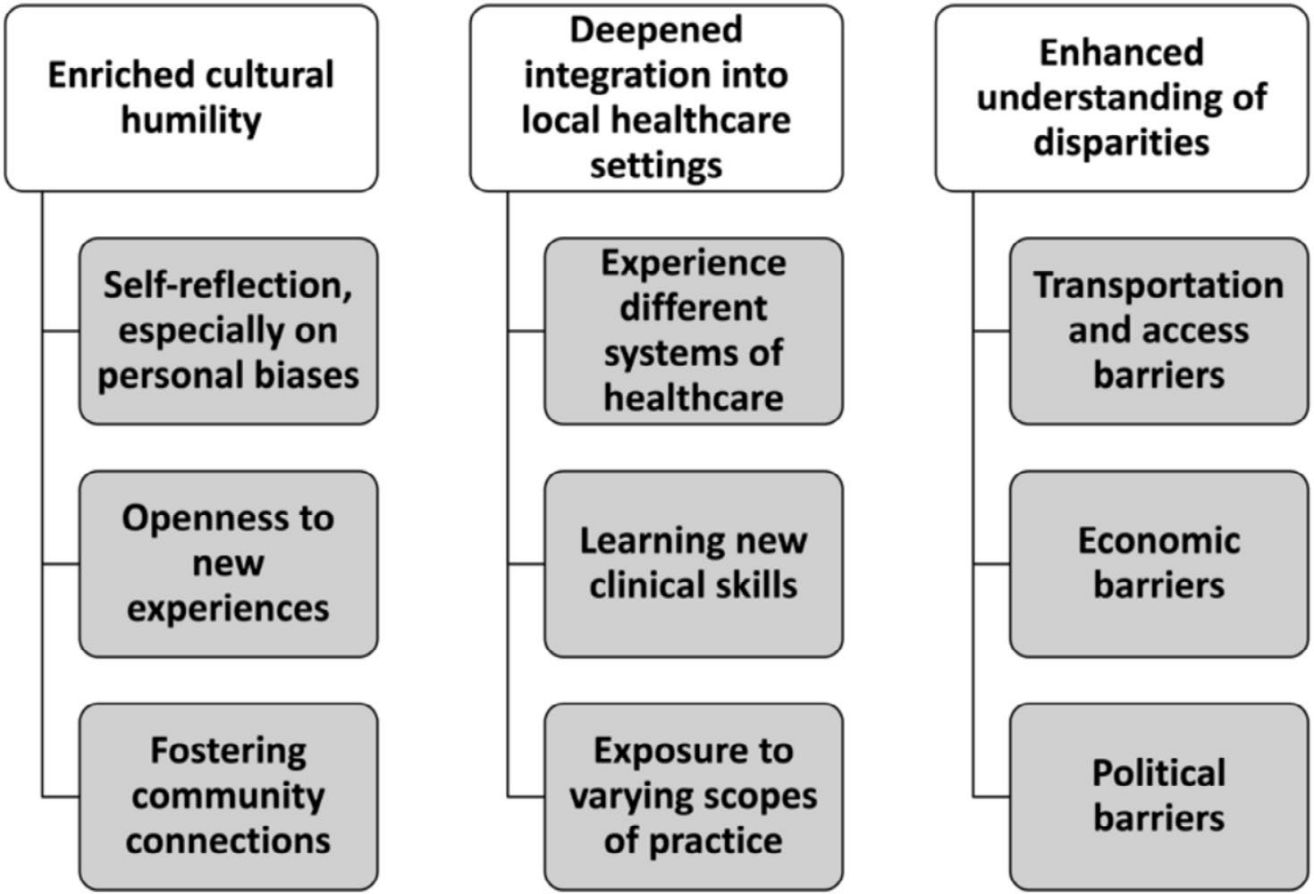
Participant reported GHI benefits.

Students reported that GHIs enriched their cultural sensitivity and humility, provided intimate immersion into local healthcare settings, and offered them deeper understandings of health disparities (Figure 1). Multidisciplinary GHIs also provided unique benefits for interdisciplinary collaboration and appreciation. All respondent feedback was de‐identified, and quotations were attributed to a unique participant identifier. Identifiers ending with “M” reflect a student who participated in a GHI trip to Malawi; identifiers ending with “B” reflect a student who participated in a GHI trip to Belize.

As “the process of interacting openly, self‐awarely, egoless, and self‐judgmentally when interacting with different ethnic groups or communities” cultural humility is critical for nurses' development both as students and as seasoned clinicians (Yüksel Kaçan and Özkaya 2025). Students endorsed enriched cultural humility through the opportunity for self‐reflection GHIs afforded them, especially through reflection on personal biases.

I think [it is] super important to get out into the world and see how other people are living, what their culture is like, how they spend their day‐to‐day. Because it really just provides a lot of self‐reflection…Like cultural humility. It's always good to reflect on ‘What were your biases going into this trip? How did they change?’ GHI_1_M

Respondents also suggested that GHIs strengthen cultural humility by fostering connections between trips participants and local partners and community members. A trip participant illustrated how connecting with diverse Belizean communities influenced his/her empathy and understanding of healthcare decision making.

Understanding the diverse communities that Belize has and how each of them are very different and how they see medicine as well. So you then you start to understand, ‘Oh, this is why they maybe don't see a primary care provider.’ …So it helped me put myself in their shoes. GHI_9_B

Furthermore, embedded deeply into local healthcare settings, students described experiencing new systems of medicine. This provided trip participants with a broader perspective of both medicine and what skills are available to them across the spectrum of practice settings. A student embedded into the local healthcare system of Malawi described the opportunity to learn new obstetrics skills while partnered with a Malawian nurse.

I did learn skills that I think we would never be taught here. We were able to measure and predict gestational age based off of feel. And stuff that you would never do that here because we have ultrasounds. I did learn stuff, even though it might not be exactly what our practice is. GHI_2_M

Finally, students depicted GHIs shaping their understandings of social determinants of health (SDOHs) and disparities in suboptimal health outcomes. In rural clinical placements, participants witnessed the impact of healthcare deserts and transportation limitations. Following spending time in a rural Malawian health setting, a respondent illustrated the barrier that limited transportation creates for patients seeking healthcare.

[Patients] had to walk hours just to get to the free clinic, or some people were so far away that it was not feasible at all … Anything that they needed, healthcare wise, they would have to wait until that one day [the mobile clinic] came because of transportation, general infrastructure, disadvantages. GHI_6_M

### 5.2 Impacts on Current and Future Practice

Participants characterised GHIs influencing their future clinical practice and the care they provide to patients. Specifically, immersion trips influenced students' desire to respect patients' cultural needs, ability to communicate in unfamiliar environments, understanding of holistic care, and motivation to promote health equity.

Respondents described GHI trips enhancing their preparedness to respect patients' cultural needs. A trip participant illustrated how the GHI influenced his/her understanding and appreciation of culturally congruent care, highlighting the unique impact a GHI has on cultural humility and respect, as compared to traditional classroom learning.

They teach cultural competency, but they don't really. You don't experience it. So you don't really understand how much someone's culture can truly impact their healthcare and their healthcare choices until you're well immersed in it. And then you're like, ‘Oh, this actually is a huge deal to them. I should respect it.’ GHI_4_B

GHIs also strengthened respondents' interpersonal clinical communication skills. In the presence of cultural communication differences, GHIs encouraged students to leverage both verbal and non‐verbal communication skills to best communicate with patients. A student reported how this experience prepared him/her to adapt the communication to patient‐specific needs and preferences.

We just were more intentional with our speech…Trying to convey that message through cultural and all the other barriers. Trying to use nonverbal communication. Try to convey that I'm not just saying these words, I'm trying to show you through nonverbal communication. GHI_7_B

When asked about their experience of healthcare in a resource limited setting, students responded that global health immersions helped them see and treat patients more holistically in clinical practice. Enhancing participants' understanding of social determinants of health and disparities, participants endorsed recognising disparities' impacts and how holistic assessment is required to address them.

You need to see the patient as a whole. I know we try to focus on this a little bit in nursing school, but when this patient comes to the hospital, you, for the most part, have no idea what's going on at home. And your home has a big impact on your health. Say you're living close to a factory, you're living with a lot of smog. All of that stuff plays into your health, and it's super important to see your patient as a whole. What they're doing, where they've been, what their life experiences are, and what their culture is especially. GHI_1_M

Respondents' understanding of disparities also impacted their desire to mitigate disparities. In the context of clinical practice, this extended not just to conceptualising the impacts of disparities but to creating care plans that mitigate their impact. A participant expounded upon the impact of GHI on his/her intention to promote health equity.

Being very mindful about resources that patients have … Maybe even having a conversation with the patients and engaging them more about, “What are some barriers that you might have financially or transportation‐wise? What is realistic for you when we're thinking about your plan of care?” GHI_8_B

Participants' experiences on global health immersion trips leave lasting impacts on their future clinical practice. Following GHI participation, students report being galvanised to provide culturally sensitive care, strengthening communication skills, recognising the importance of holistic care, and desiring to promote health equity.

### 5.3 Challenges

Participants endorsed a variety of difficult experiences while partaking in a GHI, including communication challenges in the presence of a language barrier, ethical concerns related to the trip and trip activities, and insufficient anticipatory guidance.

A common challenge encountered by students on global health immersion trips was communication in the presence of language and cultural barriers. Trip participants described a lack of adequate interpretation support. This occasionally created a reliance on fellow students who were fluent in the local language but not certified medical interpreters, posing safety concerns.

A safety issue for the patient…He was talking about chest pain. And so that's already a complicated discussion trying to discern if it's just acid reflux. And so we had a really difficult time with that. And that's something where you don't want to have a language barrier. GHI_5_B

Another challenge respondents identified was concern regarding trip ethics. A graduate student acting in the provider role raised the concern of discrepancies between medical tests conducted on the trip and the GHI team's ability to follow‐up on the results. Obtaining critical lab results without actionable interventions caused students to question the moral obligations of those conducting the test. The trip participant emphasised this challenge by describing an encounter with a hyperglycemic patient.

That's critically high at work, but we don't have insulin to give you. We're going to have to just send you back to your primary and hopefully have your medications changed … That felt bad…We're not able to actually do anything about a high blood sugar. Why are we checking it? GHI_3_B

Participants also cited ethical concerns related to trip activities. Students identified that not all trip activities were clearly salient to the trip's objectives. Certain activities, such as transient bonding with children, provided limited benefit to the communities students visited. A participant typified the harms of orphanage visits not aligned with the trip's intended purpose.

There was no real nursing aspect to going to the orphanage. It was more just ‘Oh we get to play with children.’ And I know that orphanages and stuff are a very big part of volun‐tourism… Forming those connections, I do think it was very challenging for the children. GHI_2_M

A final challenge students identified was a lack of anticipatory guidance, describing multiple deficits in their pre‐trip preparations. Respondents noted a misalignment of their pre‐departure curriculum exposure and the clinical rotations experienced on the trip. Not having pre‐trip education in all the topic areas salient to the immersion, students felt they lacked the background knowledge necessary to fully understand and appreciate their clinical experiences.

Labor and delivery type stuff was a good portion of things we either saw or had the potential to see. And a lot of us kind of just had no actual background knowledge for nursing specifically. So I feel like we couldn't quite get as much out of it just because it we didn't have any background knowledge to apply to what we were seeing. GHI_6_B

Participants highlighted inadequate review for the procedures and equipment they were expected to utilise with patients. On immersion trips that supplied their own clinical materials, such as medications, pregnancy tests, and glucometers, participants expressed frustration over limited preliminary training.

Even the glucometer I had to totally refresh my memory on. And thankfully one of the clinical faculty really knew how to use it…You don't want to prick a patient's finger twice. That was a little frustrating. GHI_5_B

A final area of inadequate anticipatory guidance described was moral injury. GHI participants noted a lack of guidance on emotionally contending with care deficits secondary to resource limitations. A student questioned the point of GHIs when participants were “helpless” to meet patient needs.

We did palpate something in [the patient's] neck. And we were thinking it was a lymph node, and it was not mobile and was a little bit suspect … The only available ultrasound was two hours away in Belize City, and he'd have to pay for it…You kind of feel a little bit helpless … It just kind of feels, ‘Is this even helpful at this point?’ GHI_8_B

Students described multiple challenges associated with participating in global health immersion including communication with language and cultural barriers, ethical concerns, and inadequate anticipatory guidance.

### 5.4 Suggestions

**FIGURE 2:**
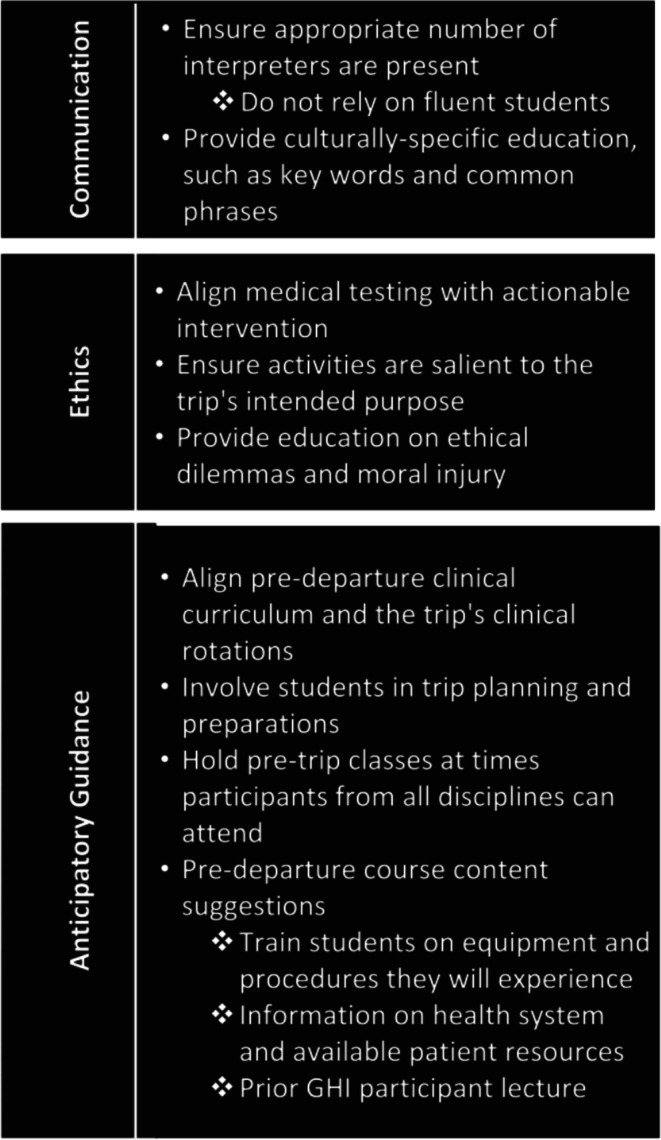
Participant reported GHI domains of challenge and suggestions.

To optimise communication, students emphasised the importance of ensuring an adequate number of interpreters is present to safely provide care to patients (Figure 2). Interpreters not only directly interpret the language but also bridge the gap of cultural considerations, such as diagnoses not commonly used or known in the U.S. Additionally, recognising the multiple facets of communication, a student described how pre‐trip education should be culturally specific and representative of the community students will interact with, such as commonly used phrases.

I think it would have been better if we just would have learned more about Malawian culture in general … On the continent, terms are very familial. Like it's not uncommon for someone to say like, ‘oh, hey, sister,’ if they don't know my name … For a lot of other people that was kind of a shock to them because it is very different than here. GHI_1_M

Respondents also noted the importance of pre‐departure education on ethical dilemmas and moral injury. Participants questioned ethical aspects of the experience and felt especially ill equipped to cope with moral injury secondary to resource limitations.

When you can't provide the care that you want to be able to provide, or that you think that you should be able to provide, that's a moral injury. And so you can't do all these things that you would to do or that we know that we could do here there. So how do you deal with that? GHI_3_B

Ideally to help mitigate some of the potential ethical dilemmas/moral injuries, students proposed expanding pre‐departure curriculums to include systems‐focused content. This included both what resources are available to patients, as well as the overall structure of the system patients are interfacing with.

Maybe a little bit more on how Belize actually practices medicine. A little more on their structure and on how that works. I was a little surprised, but not surprised, on how basically everything gets filtered back to Belize City … Maybe a little more on essentially on how big of an inconvenience it is for someone to get advanced medical care. GHI_7_B

## 6 Discussion

### 6.1 Significance of Findings

Conversations with GHI participants identified that GHIs may positively impact nurses' future practice, exposing them to new environments, cultures, and systems of healthcare. Ziegler et al. (2012) suggest that, when an individual successfully encounters an unfamiliar challenge, this propagates attitudes of openness and curiosity. This is significant because the relinquishing of power and the avoidance of assumptions are a critical foundation of cultural humility as a concept (Kuzma et al. 2019). Furthermore, GHIs encouraged participant interactions with diverse individuals; citing enhanced communication and holistic assessment skills, students experience the broad range of differences within and between groups which are an important aspect of understanding how patients' unique cultural needs influence their healthcare (Agner 2020).

In practice, respondents felt GHIs prepared them to execute care plans that are more aware and respectful of patients' cultural needs, fostering cultural sensitivity. Some participants felt this would not be possible in the setting of a traditional classroom due to the inherently immersive nature of the study abroad global health curriculum. This highlights the unique potential of GHIs to shape culturally humble and cognizant healthcare professionals. The immersive nature of GHIs also exposed students to a diverse array of clinical settings that differ in patient populations and available resources. This uniquely positioned participants to understand the direct and vast impacts of SDOHs and disparities on patient outcomes, motivating them to actively consider SDOHs while care planning.

While respondents generally perceived GHIs as positively influencing their nursing practice, they also raised significant concerns that align with the ethical dilemmas associated with GHIs as discussed in the existing literature (McKinley Yoder et al. 2022). Respondents noted a lack of bidirectional benefit with certain trip activities; this directly conflicts with the guidance put forth in the Advocacy for Global Health Partnership's Brocher Declaration outlining principles of ethical and sustainable GHIs (Advocacy for Global Health Partnerships n.d.). Communication challenges and insufficient participant preparation also highlight significant areas of concern.

### 6.2 Application of Learnings

When considering how the identified challenges are best approached, the literature emphasises clear guidelines around students' scope of practice abroad, ensuring adequate supervision even for clinical skills considered to be within a student's practice scope (Sullivan 2019). Participants in this study highlighted times of insufficient review around the procedures and equipment they were expected to use. This area could be improved upon with increased anticipatory guidance and continual GHI evaluation and updating as appropriate. Respondents also requested pre‐departure guidance on the specific culture and health system a GHI will embed them in, as well as preparation for potential moral injuries they may sustain during the immersion. Insufficient anticipatory guidance prior to GHI departure is identified in the literature as a recurring issue with immersion programming (Modlin et al. 2021; McKinley Yoder et al. 2022; Crump et al. 2010). Institutions wishing to offer GHIs are obligated to provide participants with thoughtful and thorough pre‐departure education that includes the training guidelines set forth by the Working Group on Ethics Guidelines for Global Health Training (WEIGHT) (Crump et al. 2010). We recognise the dynamic nature of these immersions and the unpredictable nature of all the experiences that may occur; this further highlights the need for continual GHI evaluation and regular updating to meet as many anticipated needs as possible.

Participants also typified the importance of adequate interpretation support and the safety risks poor support poses. Communication barriers are also key obstacles to ensuring care provided by GHI participants is effectively relayed to local healthcare providers as needed after visiting students depart the community (Zientek and Bonnell 2020). Ensuring certified medical interpreters are present and available for patient encounters is a crucial responsibility of GHI coordinators.

GHI coordinators must also consider the alignment of GHI activities with the trip's intended purpose. Volun‐tourism poses a threat to the communities students visit, regardless of amicable intentions by GHI leaders (Sullivan 2019). Bidirectional exchange can help ensure activity alignments with trip aims; for example, coordinators may consider inviting medical providers and healthcare students from the local community to take on a leadership role in GHI or participate regularly in GHI undertakings (Roebbelen et al. 2018; Kasper et al. 2020; Advocacy for Global Health Partnerships n.d.). Further, relationships with local providers, communities, and students should be longitudinal and sustained over time, allowing their perspectives to form the foundation of the GHI (Kasper et al. 2020).

### 6.3 Next Steps

There is limited literature on GHI challenges, especially ethics, from the global partners GHI programs collaborate with; this highlights the critical next steps of this project (McKinley Yoder et al. 2022). Future research should invite feedback from the international communities that students collaborate with during GHIs to understand the impact of programming on host sites and ensure mutual benefit. All stakeholder feedback (student and community members alike) is and will be considered with updating and developing immersions at our institution, and we recognise the essential importance of this feedback with gratitude for those providing it.

### 6.4 Strengths and Limitations

This work features the perspectives of future and current nurses alike, giving depth, breadth, and diversity of clinical experiences to the voices highlighted. Although the concepts of representative sampling and generalisability are debated in qualitative research, the thematic saturation this study achieved is a generally accepted measure of qualitative sampling (Robinson 2014). Also, the power differential between respondents and the interviewer was lessened by having a trained graduate student conduct the interviews; this likely bolstered candour and honesty in participants' responses. For limitations, this work notably fails to include the perspectives of international community collaborators, meaning the conclusions drawn are not representative of all stakeholders involved in GHI programmes. Also, all respondents in this study participated in GHIs at one school, meaning GHI participants at other institutions may vary in their perspectives. A small sample size limits the generalisability of the results. These limitations emphasise the importance of further research in the domain of GHI.

## 7 Conclusion

GHIs have the potential to advance culturally sensitive and respectful nursing care, as well as promote equity in healthcare. These are critical priorities in nursing education and make GHIs a powerful tool for nursing institutions of higher education. However, GHIs are also a fallible tool, requiring thoughtful planning, intentional execution, and continual evaluation. GHI alumni provide valuable perspectives not just on their GHI experiences but also the longitudinal impacts of GHI participation on nursing practice. In conjunction with evidence‐based recommendations for ethical programming, alumni's suggestions for GHI improvements can be leveraged both locally and more broadly across multiple nursing institutions. Future work should highlight the perspectives and suggestions of international community stakeholders.

## Author Contributions

C.H. and K.C. made substantial contributions to conception and design, or acquisition of data, or analysis and interpretation of data, involved in drafting the manuscript or revising it critically for important intellectual content, given final approval of the version to be published. Each author should have participated sufficiently in the work to take public responsibility for appropriate portions of the content, agreed to be accountable for all aspects of the work in ensuring that questions related to the accuracy or integrity of any part of the work are appropriately investigated and resolved.

## Disclosure

Permission to reproduce material from other sources: No third‐party materials requiring permission were used in this study.

## Ethics Statement

Any data utilised in this manuscript have been lawfully acquired in accordance with The Nagoya Protocol on Access to Genetic Resources and the Fair and Equitable Sharing of Benefits Arising from Their Utilisation to the Convention on Biological Diversity. This study was conducted as part of a quality improvement initiative evaluating global health immersion programming. Institutional Review Board (IRB) was not required, as the project focused on program evaluation for the purposes of institutional‐level improvements. Participants provided explicit consent prior to participation and all data was de‐identified to ensure participant anonymity outside of the study team.

## Consent

The authors have nothing to report.

## Conflicts of Interest

The authors declare no conflicts of interest.

## Data Availability

De‐identified data may be available from the corresponding author upon reasonable request.
